# CD5-positive marginal zone B-cell lymphoma of the mucosa-associated lymphoid tissue (MALT) of the lung

**DOI:** 10.1186/1746-1596-7-16

**Published:** 2012-02-14

**Authors:** Tadashi Terada

**Affiliations:** 1 Department of Pathology, Shizuoka City Shimizu Hospital, Shizuoka, Japan

**Keywords:** Lymphoma, lung, histopathology

## Abstract

CD5-positive marginal zone B-cell lymphoma of the mucosa-associated lymphoid tissue (MALT) of the lung is very rare. An 82-year-old Japanese woman was found to have an abnormal lung shadow on chest X-ray photography, and was admitted to our hospital. Imaging modalities including X-ray photography, computed tomography, and magnetic resonance imaging showed a small (2 × 1 × 1 cm) opacity of the right upper lobe. Transbronchial lung biopsy was performed. It showed severe proliferation of small lymphocytes. The small lymphocytes were centrocytes-like, and minor plasma cell differentiation was recognized. Lymphoepithelial lesions were scattered. Immunohistochemically, the tumor cells were positive for CD5, CD20, CD43, CD45, CD79α, bcl-2, and κ-chain, but negative for CD2, CD3, CD10, CD21, CD23, CD35, CD45RO, CD56, IgA, IgG, IgM, IgD, λ-chain, TdT, and cyclin D1. The Ki-67 labeling was 10%. CD3-positive and CD45RO-positive inflammatory T-cells were scattered in small amount. The pathological diagnosis was CD5-positive marginal zone B-cell lymphoma of the mucosa-associated lymphoid tissue (MALT) of the lung. The patient was treated with chemotherapy (CHOP: cyclophosphamide, hydroxydaunorbicin, vincristine, and predonisone), and the lung tumor disappeared. The patient is now free of the lymphoma 10 years after the first manifestation.

**Virtual slides:**

The virtual slide(s) for this article can be found here:

http://www.diagnosticpathology.diagnomx.eu/vs/1541653085652296

## Introduction

Malignant lymphoma of the lung is very rare [1]. Although any types of malignant lymphomas can occur in the lung, approximately 70-90% of the pulmonary lymphoma is marginal zone B-cell lymphoma of the mucosa-associated lymphoid tissue (MALT) of the lung [1]. Pulmonary lymphomas accounted for only 0.5% of all pulmonary neoplasms [1]. Patients with marginal zone B-cell lymphoma of the mucosa-associated lymphoid tissue (MALT) (abbreviated hereafter as MALT lymphoma) of the lung tend to be in their fifth, sixth, or seventh decades, with a slight male preponderance [1]. Etiologically, pulmonary MALT lymphoma is thought to arise in acquired MALT secondary to inflammatory or autoimmune process. The prognosis of pulmonary MALT lymphoma is relatively good when surgical resection is possible, while it may be worse in surgically-unresectable cases [1]. The 5-year survival of pulmonary MALT lymphoma is 84-94% [1]. Pulmonary MALT lymphoma progresses into diffuse large B-cell lymphoma in a small percentage, as is the case with MALT lymphoma of other organs. Other relatively common lymphomas and related diseases of the lung are diffuse large B-cell lymphoma, lymphomatoid granulomatosis, and Langerhans cell histiocytosis [1]. Histopathologically, pulmonary MALT lymphoma is an extranodal marginal zone lymphoma comprising morphologically of heterogeneous small B-cells, monocytoid cells, small lymphocytes, and scattered immunoblasts-like and centroblasts-like cells. There is a plasma cell differentiation in a proportion of cases. The neoplastic cells typically infiltrate into the bronchial mucosal epithelial cells, creating lymphoepithelial lesions [1].

Most of MALT lymphoma is negative for CD5 [2]. However, there are a few reports of CD5-positive MALT lymphoma of the lung and other organs [3-13]. The CD5 positivity in MALT lymphoma made the diagnosis difficult, and many differential diagnoses should be considered. The significance, mechanism, and biological behaviors of CD5-positive MALT lymphoma are unknown [3-13]. The author herein reports the case of a CD5-positive pulmonary MALT lymphoma with good prognosis.

## Case report

**Figure 1:**
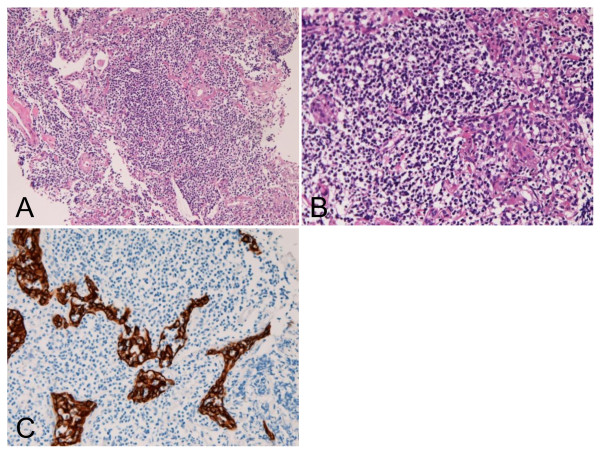
**Histological features**. A: Diffuse atypical lymphoid cell proliferation is seen. HE, ×100. B: The atypical cells are small lymphoid cells with hyperchromatic nuclei. Lymphoepithelial lesions are seen. HE, × 400. C: The lymphoepithelial lesions are clearly accentuated by cytokeratin immunostaining. AE1/3 immunostaining, ×200.

An 82-year-old Japanese woman was found to have abnormal lung shadow on chest X-ray photography at a private hospital. She was referred to our hospital for scrutiny. Imaging modalities including X-ray photography, computed tomography and magnetic resonance imaging showed a small (2 × 1 × 1 cm) opacity of right upper lobe. Abnormal blood laboratory data included mild leukocytosis (9.5 × 10^9 ^/L; normal 3.5-9.0 × 10^9^/L), anemia (367 x10^10 ^/L; normal, 370-480 × 10^10^/L; hemoglobin 9.5 g/dl, normal 11 g/dl-16 g/dl), decreased total protein (63 g/L; normal 65-92 g/L), low zinc turbidity test (2.3 U; normal 4.0-12.0 U), and increased blood uria nitrogen (2.4 μmol/L; normal 2.9-8.9 μmol/L). The white blood cell compartment was as follows: basophils 1%, band neutrophils 2% (low), segmented neutrophils 84% (high), and lymphocytes 11% (low). Eosinophils and precursor cells were not recognized. Other data were normal. There was no M-protein. No hyper-gamma-globulinemia was noted. Examination of serum immunoglobulin components was not performed. Transbronchial lung biopsy (TBLB) was performed. The TBLB specimens consisted of several fragments. They are fragments of the proliferated lymphocytes (Figure 1A). The TBLB showed severe proliferation of small lymphocytes with scattered small centroblast-like cells (Figure 1B). The lymphocytes were centrocytes-like, and minor plasma cell differentiation was recognized (Figure 1B). Lymphoepithelial lesions were scattered (Figure 1B), and they were highlighted by cytokeratin immunostaining (Figure 1C). No follicular structures were found. No findings of Burkitt lymphoma were recognized.

An immunohistochemical study was performed with the use of Dako's EnVision method (Dako Corp, Carpinteria, CA), as previously described [14-16].

Each immunostaing was pretreated by microwave oven heating for 10 minutes. The antibodies, clones, and dilution used were as follows: CD2 (Dako, MT910, 1:100), CD3 (Dako, F7.2.38, 1:25), CD5 (Dako, CD5/54/F6, 1:50), CD10 (Novocastra, New Castle Upon Tyne, UK, NCL-CD10-270, 1:100), CD20 (Dako, L26, 1:100), CD21 (Dako, 1F8, 1:25), CD23 (Dako, MHM-6, prediluted), CD35 (Dako, Ber-MAC-DRC, 1:25), CD43 (Dako, DF-T1, prediluted), CD45 (Dako, LCA, 1:100), CD45RO (Dako, UCHL-1, 1:100), CD56 (Novocastra, NCL-CD56-1B6, 1:100,), CD79α (Dako, JCB117, 1:200), bcl-2 (Dako, 124, 1:150), cyclin D1 (Dako, DCS-6), TdT (Dako, A3524, 1:100), IgA (Dako, 6E2C1, 1:100), IgG (Dako, A57H, 1:150), IgM (Dako, polyclonal, 1:50), κ-chain (Dako, polyclonal, 1:200), λ-chain (Dako, polyclonal, 1:100), and Ki-67 (MBL Japan, MIB-1, 1:200)

**Figure 2:**
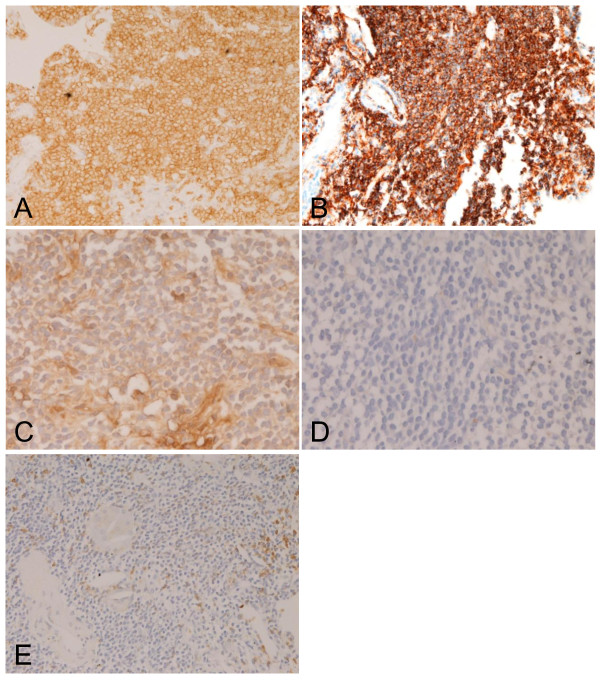
**Immunohistochemical features**. The tumor cells are positive for CD5 (A), CD20 (B), and κ-chain (C). λ-chain was negative (D). A, B, C: ×200. D, x400. The tumor cells were negative for CD3 (E). A small amount of CD3-positive inflammatory cells are seen (E). E: ×200.

Immunohistochemically, the tumor cells were positive for CD5 (Figure 2A), CD20 (Figure 2B), CD43, CD45, CD79α, bcl-2, and κ-chain (Figure 2C), but negative for λ-chain (Figure 2D), CD2, CD3 (Figure 2E), CD10, CD21, CD23, CD35, CD45RO, CD56, TdT, IgA, IgG, IgM, IgD, and cyclin D1. The Ki-67 labeling was 10%. CD3-positive and CD45RO-positive inflammatory T-cells were scattered in a small amount. Light chains immunostainings showed light chain restriction (Figures 2C and 2D) and a small number of plasma cells (Figure 2C). Methylgreenpyronine staining showed a small number of plasma cells. The pathological diagnosis was CD5-positive MALT lymphoma. After the TBLB, a bone marrow biopsy was performed. It showed normocellular marrow with normal erythroid, granulocytoid and megakaryocytic maturation. No atypical cells were identified in the bone marrow.

The patient rejected surgical procedure because of very old age, and was treated with chemotherapy (CHOP: cyclophosphamide, hydroxydaunorbicin, vincristine, and predonisone), and the lung tumor disappeared. The patient is now free of MALT lymphoma 10 years after the first manifestation.

## Discussion

The present lung tumor was composed of B cells. The lymphoid cells were small. Plasma cell differentiation was seen in a slight degree. Lymphoepithelial lesions were scattered. κ-chain was positive but λ-chain was negative, confirming the light chain restriction and a tumor cell monoclonality. Therefore, the present case was low-grade B-cell lymphoma. The low grade and intermediate grade B-cell lymphoma consists of small lymphocytic lymphoma (low grade), lymphoplasmcytic lymphoma (low grade), follicular lymphoma (low grade), MALT lymphoma (low grade), and mantle cell lymphoma (intermediate grade). The present case is not small lymphocytic lymphoma/chronic leukocytic leukemia, which shows monotonous proliferation of mature small lymphocytes and free from centrocyte-like cells and lymphoepithelal lesions. Immunohistochemically, small lymphocytic lymphoma expresses IgM/IgD, CD20, CD22, CD5, CD19, CD79a, CD23, CD43 and CD11c. CD10 is negative. In the present case, IgM/D and CD23 were negative, supporting that the current case is not small lymphocytic lymphoma. The current case is not lymphoplasmacytic lymphoma because the plasma cells were scant in number and there was no M-protein. Negative immunoglobulins except for κ-chain in tumor cells are also against the diagnosis of lymphoplasmacytic lymphoma. The presence of centrocyte-like cells, lymphoepithelial lesions, and plasmacytoid differentiation is characteristic features of MALT lymphoma. The clinical features are also against lymphoplasmacytic lymphoma, which frequently shows extrapulmonary and marrow involvements and shows M-protein and hyper-gamma-globulinemia. Positive κ-chain in the current case may be due to the plasmacytoid differentiation of MALT lymphoma. The plasma cells in the present case are due to plasmacytoid differentiation of the MALT lymphoma, which is a characteristic feature of MALT lymphoma [2]. The present tumor is not follicular lymphoma because no follicular structures were recognized. The positive reaction for CD5 and negative reaction for CD10 and CD35 also exclude the possibility of follicular lymphoma, in which CD5 is negative and CD10 and CD35 (a marker of follicular dendritic cells) are positive. Although CD10 is infrequently positive in follicular lymphoma, the morphology, immunohistochemistry, the presence of plasmacytoid cells and lymphoepithelial lesions are in favor of MALT lymphoma rather than follicular lymphoma. The present tumor is not mantle cell lymphoma, because of negative cyclin D1 which is almost always positive in mantle cell lymphoma [17]. The differential diagnosis of CD5-positive MALT lymphoma and cyclin D1-negative mantle cell lymphoma is very difficult. Mantle cell lymphoma is composed of monotonous proliferation of small cells of mantle zone. In mantle cell lymphoma, no centrocyte-like cells, plasma cell differentiation, or lymphoepithelial lesions are seen. In the present case, the histologies are those of MALT lymphoma. In addition, the good prognosis of the present case is in favor of MALT lymphoma rather than mantle cell lymphoma, which shows poor prognosis. Therefore, the author diagnosed this tumor as MALT lymphoma. A genetic study of t(11:18)(q21:q21) (MALT lymphoma) and t(11:14)(q13:q31)(mantle cell lymphoma) are required for the distinction. In the present case, no genetic study was performed. The present case is different from benign lymphoproliferative diseases of lung, such as follicular bronchiolitis, lymphocytic interstitial pneumonia, and nodular lymphoid hyperplasia [18] because the present case shows neoplastic characteristics which were demonstrated by B-cell monoclonality and light chain restriction (light chain monoclonality). Thus, the neoplastic characteristics of the present case rule out these reactive lymphoproliferative conditions [18]. These findings are compatible with a diagnosis of MALT lymphoma of the lung.

Immunohistochemically, the low and intermediate grade B-cell lymphomas show the following immunophenotypes: follicular lymphoma, CD5-, CD10 +, CD23 -, CD43 -, cyclin D1 -; MALT lymphoma, CD5-, CD10-, CD23-, CD43+/-, cyclin D1 -; mantle cell lymphoma, CD5+, CD10-, CD23-, CD43 +/-, cyclin D1 + [2]. The present case was as follows: CD5+, CD10-, CD43+, cyclin D1 -. The immunoprofile is compatible with to MALT lymphoma. The histological features of the present case, such as lymphoepithelial lesions and plasma cell differentiation, are also those of MALT lymphoma [2].

The CD5 positivity of the present MALT lymphoma is unique. CD5-positive MALT lymphoma has been rarely reported [3-13]. The brief clinicopathological findings of the present case and reported cases are summarized in Table 1. Ballesteros et al [3] mentioned that CD5-positive MALT lymphoma tended to show wide spread disease in the literature, and presented three cases of CD5-positive MALT lymphoma and stated that CD5-positive MALT lymphomas were localized tumors. The sites of the lymphoma were uterus, lymph nodes, lung, and conjunctiva [3]. Tasaki et al [4] reported two cases of CD5-positive MALT lymphoma of ocular adnexa. They stressed the differential diagnosis between CD5-positive MALT lymphoma and mantle cell lymphoma [4]. Hisabe et al [5] reported a case of CD5-positive MALT lymphoma of the rectum which regressed after administration of antibiotics. Sundeen et al [6] mentioned 11 cases of CD5-positive B-cell small lymphocytic malignancies of various organs. Mikolaenko and Listinsky [7] reported a case of CD5-positive MALT lymphoma with systemic involvement and Waldenstrom syndrome. Ferry et al [8] reported 3 cases of CD5-positive MALT lymphoma of orbit and tongue with recurrence. Kubota et al [9] reported 3 case of CD5-positive MALT lymphoma of orbit with autoantibodies. Tsukamoto et al [10] reported a case of cutaneous CD5-positive MALT lymphoma resembling the plasma cell variant of Castleman's disease. Wenzel et al [11] reported a case of CD5-positive MALT lymphoma of the conjunctiva with early dissemination and aggressive clinical behavior. Batstone et al [12] reported a case of CD5-positive MALT lymphoma of the breast and lymph nodes with genetic analyses. Heuring et al [13] reported a case of conjunctival CD5-positive MALT lymphoma. Because the number of cases of CD5-positive MALT lymphoma is very small, its biological characteristics are unknown.

**Table 1:** The present case and reported cases of CD5-positive MALT lympoma

Case	Age	Sex	Site	Morphology	LEL	Immunophenotype	Reporters	**Ref**.
No.	(yr)							
1	72	F	Uterus	MALT	+	CD3 -, CD5 +, CD20 +	Ballesteros et al	[3]
1	72	F	Lymph nodes	MALT	+	CD3 -, CD5 +, CD10 -, CD11c +, CD20+, CD23 -	Ballesteros et al	[3]
2	56	M	Lung	MALT	+	CD3-, CD5 +, CD10+, CD20+,	Ballestetos et al	[3]
3	70	F	Conjunctive	MALT	+	CD3-, CD5+, CD10-, CD11c-, CD20 +	Ballestetos et al	[3]
4	57	F	Eyelid	MALT	+	CD3-, CD5+, CD10-, CD20+, CD23 - CD79α+, cyclin D1-, bcl-2+	Tasaki et al	[4]
5	67	M	Orbit	MALT	-	CD3-, CD5+, CD10-, CD20+, CD23 - CD79α+, cyclin D1-, bcl-2+, κchain+	Tasaki et al	[4]
6	70	F	Rectum	MALT	ND	CD5+, CD10-, CD20+, CD45RO-, bcl-2+	Hisabe et al	[5]
7	75	M	Systemic	MALT	+	CD5+, CD10-, CD19+, CD20+, CD11C-FMC7+, IgMλ+	Mikolaenko et al	[7]
8	77	M	Orbit	MALT	+	CD5+, CD10-, CD20+, CD22 +CD23-, CD43-, IgMλ+, Cyclin D1-, leu7 +	Ferry et al	[8]
9	44	M	Orbit, blood Marrow, lymph nodes	MALT	+	CD5+, CD10-, CD20 + CD22+, CD23-, CD43+, IgMκ+, leu8+, cyclin D1-	Ferry et al	[8]
10	62	F	Tongue	MALT	+	CD5+, CD10-, CD20+, CD23+, CD43+sIg-, cyclin D1-	Ferry et al	[8]
11	82	ND	Orbit	MALT	ND	CD3-, CD5+, CD20+, CD23-, cyclinD1-	Kubota et al	[9]
12	70	ND	Orbit	MALT	ND	CD3-, CD5+, CD20+, CD23-, cyclinD1-	Kubota et al	[9]
13	84	ND	Orbit	MALT	ND	CD3-, CD5+, CD20+, CD23-, cyclinD1-	Kubota et al	[9]
14	57	F	Skin	MALT	ND	CD3-, CD5+, CD10-, CD20+, CD21-, CD23-, CD43+, bcl-2+, BSAP+, IgGκ+	Tsukamoto et al	[10]
15	73	M	Conjunctiva	MALT	ND	CD2-, CD3-, CD5+, CD10-, CD20+, CD23-, CD79α+, Cyclin D1-	Wenzel et al	[11]
16	87	F	breast Lymph nodes	MALT	+	CD3-, CD5+, CD10-, CD21-, CD23-, bcl-2+κchain-,λchain+, bcl-6-. cyclinD1-	Batstone et al	[12]
17	79	M	Conjunctiva	MALT	-	CD5+, CD20+, CD23-, CD30-,	Heuring et al	[13]
18	82	F	Lung	MALT	+	CD2-, CD3-, CD5+, CD10-, CD20+, CD23-, CD43+, CD45+, CD45RO-, CD56-, CD79αbcl-2+,κchain+,λchain-, IgA-, IgG-, IgM-, TdT-, cyclinD1-	Terada Present case	

ND: not described. LEL: Lymphoepithelial lesion.

The MALT lymphoma of the present case disappeared after CHOP chemotherapy, and has not recurred for 10 years. Surgical procedure was not done because the patient rejected the surgical operation. The prognosis of pulmonary MALT lymphoma is not worse. The five year survival is 84-94% [1]. Kurtin et al. [19] described that lymphoma-specific survival of pulmonary MALT lymphoma was 71.7% at 10 years. Borie et al [20] described that the 5 years survival was 90% and 10 years survival was 72% in pulmonary MALT lymphoma. The present patient is alive free from tumor 10 years after the CHOP medical treatment without surgical intervention.

## Consent

Written informed consent was obtained from the patient for publication of this case report and accompanying images. A copy of the written consent is available for review by the Editor-in-Chief of this journal.

## Competing interests

The authors declare that they have no competing interests.
